# From bench to bedside: primary aldosteronism revisited

**DOI:** 10.1530/EC-26-0524

**Published:** 2026-09-10

**Authors:** Jia Wei, Tracy Ann Williams

**Affiliations:** Medizinische Klinik und Poliklinik IV, Klinikum der Universität München, Ludwig-Maximilians-Universität München, Munich, Germany

**Keywords:** adrenalectomy, aldosterone-producing adenoma, aldosterone-producing micronodule, HISTALDO classification, oxidative stress, PAMO criteria, PASO criteria, primary aldosteronism, tissue multi-omics

## Abstract

Primary aldosteronism (PA) is a common, under-recognised cause of hypertension carrying cardiovascular and renal risk beyond blood pressure alone. Management requires accurate subtype diagnosis: lateralised disease may be cured by adrenalectomy, whereas bilateral disease usually requires mineralocorticoid receptor antagonist therapy. The PASO criteria standardised postsurgical outcome assessment and showed that biochemical remission is achieved in most patients with adrenal vein sampling-confirmed lateralised disease, whereas clinical remission is variable, reflecting pre-existing hypertensive burden, age and sex. The PAMO criteria extend this framework to medically treated PA and show that complete clinical response is uncommon. At the tissue level, the HISTALDO classification distinguishes classical lateralised disease, typically caused by an aldosterone-producing adenoma, from non-classical disease, dominated by multiple micronodules; the latter carries a higher risk of postsurgical persistent or recurrent aldosteronism. These observations support a broader model in which PA forms a continuous rather than binary spectrum, from subclinical renin-independent aldosteronism and age-related micronodular remodelling to overt bilateral and lateralised disease. Tissue omics studies support a model in which aldosterone-producing lesions progress from zona glomerulosa cells to micronodules and adenomas. *KCNJ5*-mutated adenomas may follow a direct route, arising without a detectable micronodule stage, possibly because reduced oxidative stress favours early cell survival and expansion. The adenoma is therefore not a static endpoint but a maturing tissue, in which immune remodelling and changes in cell survival mechanisms may shape progression towards a hypersecretory state. Together, these findings link clinical phenotype to adrenal tissue biology and support a dynamic, genotype-modulated model of PA pathogenesis.

## Introduction

Aldosterone is the principal mineralocorticoid hormone, produced by the zona glomerulosa of the adrenal cortex, where it regulates renal sodium reabsorption and potassium excretion and thereby modulates blood volume and blood pressure. Under physiological conditions, aldosterone secretion is tightly regulated mainly by the renin–angiotensin–aldosterone system, but in primary aldosteronism (PA), one or both adrenal glands produce aldosterone relatively independently of normal regulatory control (1, 2). PA is the most common endocrine cause of secondary hypertension but remains substantially underdiagnosed even in high-risk populations (3, 4). This is clinically important because aldosterone excess causes cardiovascular and renal injury beyond the effect of blood pressure alone. Compared with patients with similarly severe essential hypertension, patients with PA have greater cardiovascular target-organ damage and a higher risk of complications, including atrial fibrillation, left ventricular hypertrophy and stroke, and show more pronounced evidence of renal injury, including albuminuria and chronic kidney disease (5, 6, 7, 8).

A key therapeutic question is whether aldosterone production is lateralised or bilateral. In lateralised PA, adrenalectomy removes the dominant source of aldosterone and offers the possibility of cure. In bilateral disease, or when surgery is not appropriate, treatment currently relies on mineralocorticoid receptor (MR) antagonists (1, 2, 9). This distinction is essential for clinical management, but it does not fully capture the underlying disease process. Clinical physiology studies have shown that renin-independent aldosterone production is not confined to overt PA but forms a graded continuum extending from normotension to severe hypertension (10). Studies of adrenal tissue provide the cellular and molecular correlate of this continuum. Ageing is associated with accumulation of discrete aldosterone-producing micronodules (APMs) in the zona glomerulosa layer and a shift towards partly renin-independent aldosterone physiology (11). In overt PA, bilateral disease may be characterised less by diffuse hyperplasia than by multiple APMs (12), whereas lateralised disease is typically associated with an aldosterone-producing adenoma (APA) (13, 14). Together, these clinical and tissue findings support a model in which PA is better understood not as a binary disorder but as a continuous spectrum of adrenal remodelling (15).

This review uses variability in treatment outcome as a clinical entry point into that biology. Differences in outcome after adrenalectomy – ranging from complete clinical and biochemical success to persistent hypertension, persistent aldosteronism or recurrence – point to biological heterogeneity that conventional clinical subtyping does not fully capture. At the tissue level, the contrast between microscopic aldosterone-producing lesions and dominant adenomas suggests that these lesions differ not only in size but also in their capacity to persist and expand and in their ability to reshape the surrounding adrenal tissue. The aim of this review is to connect the phenotype observed after treatment with the cellular processes that determine how aldosterone-producing lesions arise, persist and progress.

### Adrenal zonation and CYP11B2 immunohistochemistry

The adrenal cortex is functionally zonated, with mineralocorticoids, glucocorticoids and adrenal androgens produced predominantly in distinct concentric layers comprising the outer zona glomerulosa, the zona fasciculata and the inner zona reticularis. The zona glomerulosa is specialised for aldosterone synthesis, which depends on CYP11B2 (aldosterone synthase), the terminal enzyme that catalyses the sequential conversion of deoxycorticosterone to corticosterone, 18-hydroxycorticosterone and finally aldosterone (16). In the normal adrenal, CYP11B2 is restricted to zona glomerulosa-lineage aldosterone-producing cells. In contrast, cortisol synthesis depends on the closely related isozyme 11β-hydroxylase (CYP11B1), expressed predominantly in the zona fasciculata, while the zona reticularis is specialised for adrenal androgen production. The development of specific monoclonal antibodies against CYP11B1 and CYP11B2 therefore made it possible to distinguish cortisol-producing from aldosterone-producing cells *in situ* (17, 18). CYP11B2 immunohistochemistry has since become the cornerstone of modern adrenal histopathology in PA (19, 20), providing a functional map of aldosterone-producing tissue that enables lesion classification and supports integration with spatially resolved molecular analyses (21, 22). This functional mapping also changed how somatic driver mutations in PA are interpreted. By identifying CYP11B2-positive tissue directly, mutations can be assigned to aldosterone-producing lesions rather than to adrenal nodules defined by morphology alone (23). Thus, zonation, CYP11B2 mapping and mutation analysis provide the anatomical and molecular context for understanding why some aldosterone-producing lesions remain microscopic, whereas others expand into dominant adenomas.

### Surgical treatment outcomes

**Figure 1:**
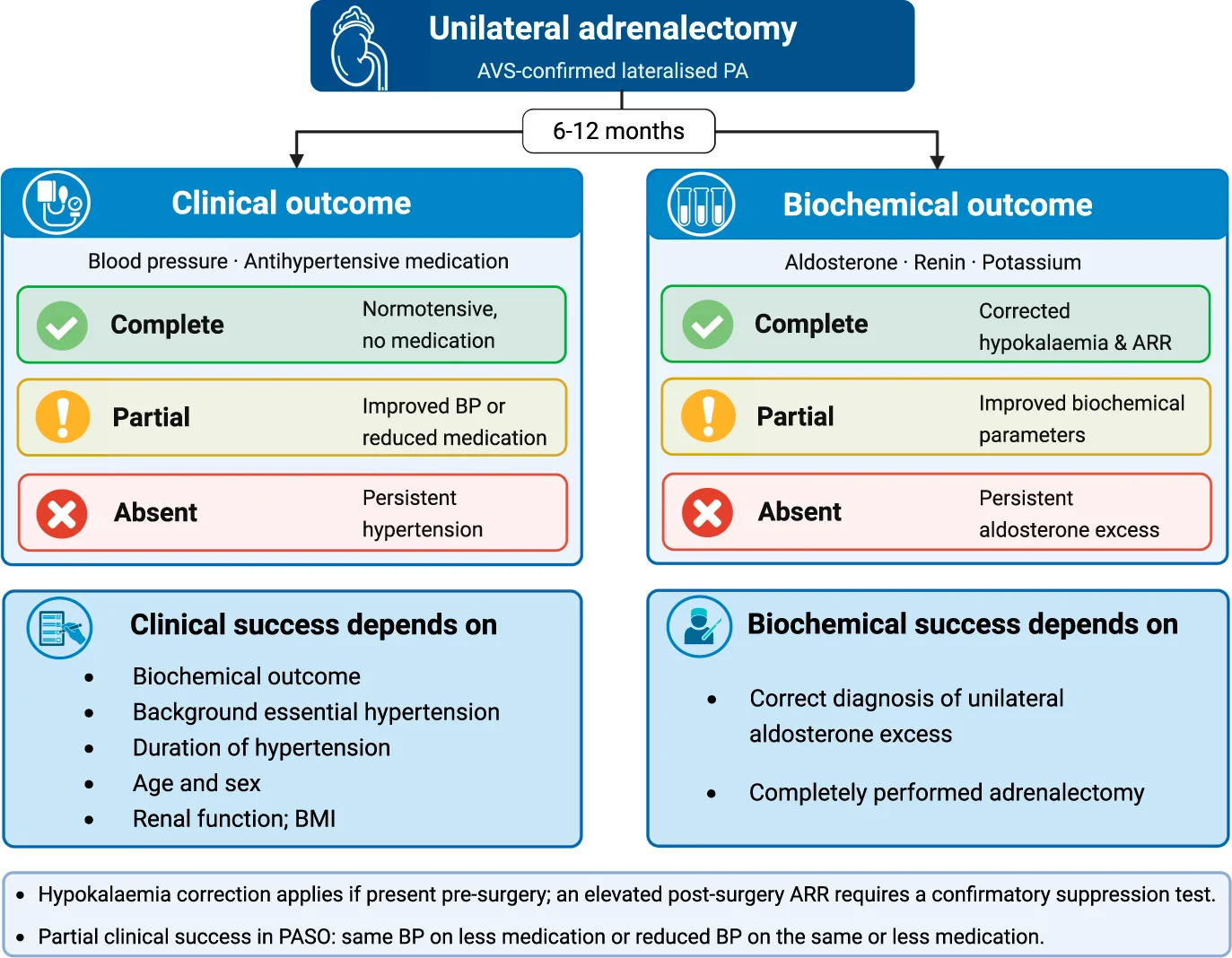
Standardised assessment of surgical outcome after adrenalectomy. Clinical and biochemical outcomes after unilateral adrenalectomy for AVS-confirmed lateralised PA are graded independently as complete, partial or absent at 6–12 months according to the PASO criteria. Clinical success depends on the biochemical outcome and on preoperative phenotype (background essential hypertension, duration of hypertension, age, sex, renal function and BMI), whereas biochemical success depends on correct diagnosis of unilateral aldosterone excess and a completely performed adrenalectomy. In the PASO criteria, hypokalaemia correction applies only where hypokalaemia was present pre-surgery, and an elevated post-surgery ARR requires a confirmatory suppression test; partial clinical success is defined as the same blood pressure with less medication or reduced blood pressure with the same or less medication. ARR, aldosterone-to-renin ratio; AVS, adrenal vein sampling; PA, primary aldosteronism; PASO, PA surgical outcome. Created in BioRender https://BioRender.com/us3cpct.

Historically, postoperative outcomes after adrenalectomy were difficult to compare because studies used variable definitions of remission (24, 25). The international Primary Aldosteronism Surgical Outcome (PASO) consensus addressed this by standardising outcome assessment in two complementary categories: clinical outcome, based on blood pressure and antihypertensive medication use, and biochemical outcome, based on aldosterone, renin and potassium, graded as complete, partial or absent success (26) (Fig. 1).

Applied to 705 patients from 12 referral centres in nine countries, the PASO criteria showed complete biochemical success in 94% of patients, confirming that adrenal vein sampling (AVS)-guided adrenalectomy reliably removes the dominant source of aldosterone overproduction in appropriately selected patients with lateralised PA (26). Complete clinical success was less frequent and remained variable: 37% showed normal blood pressure without antihypertensive medication, although a further 47% had partial clinical success, so that most patients benefited clinically even when complete clinical remission was not achieved.

The distinction between clinical and biochemical outcomes is biologically informative, not just useful for standardising postoperative assessment. Complete biochemical success principally reflects accurate lateralisation and surgical removal of the dominant aldosterone-producing source. In contrast, clinical success depends partly on biochemical success but also reflects the preoperative phenotype. Shorter duration of hypertension, female sex, lower antihypertensive medication burden, lower body mass index, absence of target-organ damage and imaging features of a dominant adrenal lesion are associated with complete clinical success (26, 27). Between-centre variation in clinical remission can therefore be partly attributed to differences in patient selection, cohort demographics and baseline hypertensive disease burden.

PASO also made incomplete biochemical success clinically interpretable. In some patients, persistent aldosteronism after adrenalectomy may reflect asymmetrical bilateral PA, in which one adrenal dominates sufficiently to lateralise on AVS, but the contralateral gland also contributes to autonomous aldosterone production. Removing the dominant gland may therefore reduce aldosterone excess without necessarily abolishing it. This interpretation is supported by tissue studies of persistent PA after AVS-guided adrenalectomy. In an international cohort of 283 patients who underwent unilateral adrenalectomy on the basis of strong AVS lateralisation, 16% did not achieve complete biochemical success of PA postoperatively. Among those with persistent disease, 73% had multiple CYP11B2-positive areas in the resected adrenal (28). Thus, multiple aldosterone-producing areas within the resected dominant gland suggest a multifocal adrenal process and raise the possibility that similar, although less active, lesions may persist in the contralateral adrenal. Consistent with this interpretation, patients with partial or absent biochemical success after adrenalectomy had lower lateralisation indices (29, 30) and higher presurgical contralateral aldosterone secretion (13), suggesting that incomplete biochemical success often reflects a less clearly lateralised disease phenotype.

Although adrenalectomy is conventionally reserved for lateralised PA, several small cohorts suggest that surgery may benefit carefully selected patients with AVS-defined bilateral disease (31, 32, 33). In a single-centre cohort, 40 patients with bilateral PA underwent unilateral adrenalectomy, usually because blood pressure remained inadequately controlled or MR antagonists were not tolerated. The adrenal selected for removal was either the dominant gland on AVS or the gland with the greatest morphological abnormality on imaging; hypertension was cured in 15% and improved in a further 20% (31). An international proof-of-principle cohort subsequently assessed 56 surgically treated patients with AVS-defined bilateral PA from six referral centres, including 43 treated by unilateral adrenalectomy and 13 by bilateral adrenal surgery (32). Most patients derived clinical benefit, defined as complete or partial clinical success, and histopathology showed discrete aldosterone-producing lesions, including APAs, multiple APMs and diffuse hyperplasia. Among bilaterally resected adrenal pairs, the aldosterone-producing lesions were often similar in both adrenals, consistent with a bilateral process (32). Comparable benefit, including improved blood pressure control, reduced antihypertensive burden and resolution of hypokalaemia, has been reported in a further single-centre series (33).

A related question is whether contralateral aldosterone production should itself preclude surgery when lateralisation is otherwise clear: in a single-centre series of 29 patients who lateralised on AVS despite an elevated contralateral suppression index, unilateral adrenalectomy achieved complete or partial clinical success in approximately three-quarters, albeit over short follow-up (34). Whether contralateral aldosterone production undermines surgical benefit appears to depend on the degree of lateralisation: a lower lateralisation index or greater contralateral secretion is associated with worse outcomes, whereas contralateral production need not preclude benefit when lateralisation is otherwise strong.

Together, these studies suggest that adrenalectomy can produce meaningful clinical and biochemical benefit in selected patients with bilateral or asymmetrical bilateral PA. A proposed mechanism is that, in milder disease with a relatively low level of autonomous aldosterone production, removal of one adrenal gland may reduce autonomous aldosterone secretion sufficiently to restore aldosterone to a range appropriate for the patient’s salt and volume status (31).

### Medical treatment outcomes

Medical therapy is recommended for bilateral PA and for patients with lateralised PA who do not undergo surgery. In a retrospective cohort with comparable cardiovascular risk profiles and blood pressure, patients treated with an MR antagonist for PA had a higher risk of incident cardiovascular events, mortality, diabetes and atrial fibrillation than those with essential hypertension; notably, the excess risk of cardiovascular events and mortality was limited to patients whose renin activity remained suppressed (<1 ng/mL per h) on treatment, whereas patients treated with higher MR antagonist doses who achieved unsuppressed renin showed no significant excess risk (35). The same renin-dependent pattern is seen for incident atrial fibrillation: excess risk relative to essential hypertension was confined to MR antagonist-treated patients whose renin remained suppressed, whereas those whose renin increased and those treated by adrenalectomy showed no significant excess (36). Persistent renin suppression therefore appears to reflect residual mineralocorticoid excess, providing a rationale for biochemically guided treatment targets.

The need for standardised medical outcome criteria led to the Primary Aldosteronism Medical Treatment Outcome (PAMO) consensus, which extended the PASO logic to targeted medical therapy by defining complete, partial and absent biochemical and clinical responses (37). Complete biochemical response requires correction of hypokalaemia (if present pre-treatment) and reversal of renin suppression, whereas complete clinical response requires normalised blood pressure without additional antihypertensive medication (i.e. on MR antagonist monotherapy). Applied to an international cohort of 1,258 patients, PAMO showed that, among those with follow-up data, complete biochemical response was achieved in only 53% and complete clinical response in fewer than one in five; suboptimal targeted dosing, driven specifically by spironolactone dose rather than the combined MR antagonist defined daily dose, was a major modifiable factor, while independent predictors of complete clinical response paralleled the surgical cohort, including female sex, lower baseline antihypertensive requirement and absence of target-organ damage (37). Real-world data from the SPAIN-ALDO registry are concordant: among 402 medically treated patients assessed after at least six months of MR antagonist or amiloride therapy, complete clinical response was achieved in 16.2% of those with clinical follow-up (*n* = 389; partial 65.5%, absent 18.3%), and complete biochemical response in 50.1% of those with biochemical follow-up (*n* = 261; partial 21.5%, absent 28.4%) (38). The same predictors recurred; favourable clinical response was associated with female sex, lower body mass index, lower baseline medication burden and higher potassium, whereas complete biochemical response was associated with lower baseline aldosterone, higher baseline renin, spironolactone rather than eplerenone and higher MR antagonist doses (38).

Together, these data support titration of medical therapy to biochemical response, particularly reversal of renin suppression. Higher MR antagonist doses improve biochemical response, but dose up-titration carries a tolerability trade-off. In a 394-patient spironolactone cohort, doses >50 mg were associated with more adverse effects, the dose-related increase reaching significance only in men, with no accompanying advantage in blood pressure control or potassium compared with a ≤50 mg regimen alongside a heavier concomitant antihypertensive load (39). Because the lower-dose group received more co-medication, particularly diuretics, which may themselves have raised renin, the comparable outcomes are better read as an argument for tolerability and adherence than as evidence that low-dose spironolactone alone achieves the biochemical target.

### Histopathology of primary aldosteronism

The tissue-level counterpart to standardised outcome measurement is standardised histopathology. AVS-guided adrenalectomy followed by CYP11B2 immunohistochemistry has shown that lateralised PA is more complex than the traditional model of a solitary APA, comprising instead a spectrum of histologically distinct lesions. This diversity helps explain why two patients can lateralise similarly on AVS yet have different biochemical and clinical outcomes after surgery. The prototypical lesion remains the APA, which tends to present with the most severe hyperaldosteronism (13, 40). Yet a substantial minority of operated patients fail to achieve biochemical remission (26).

**Figure 2:**
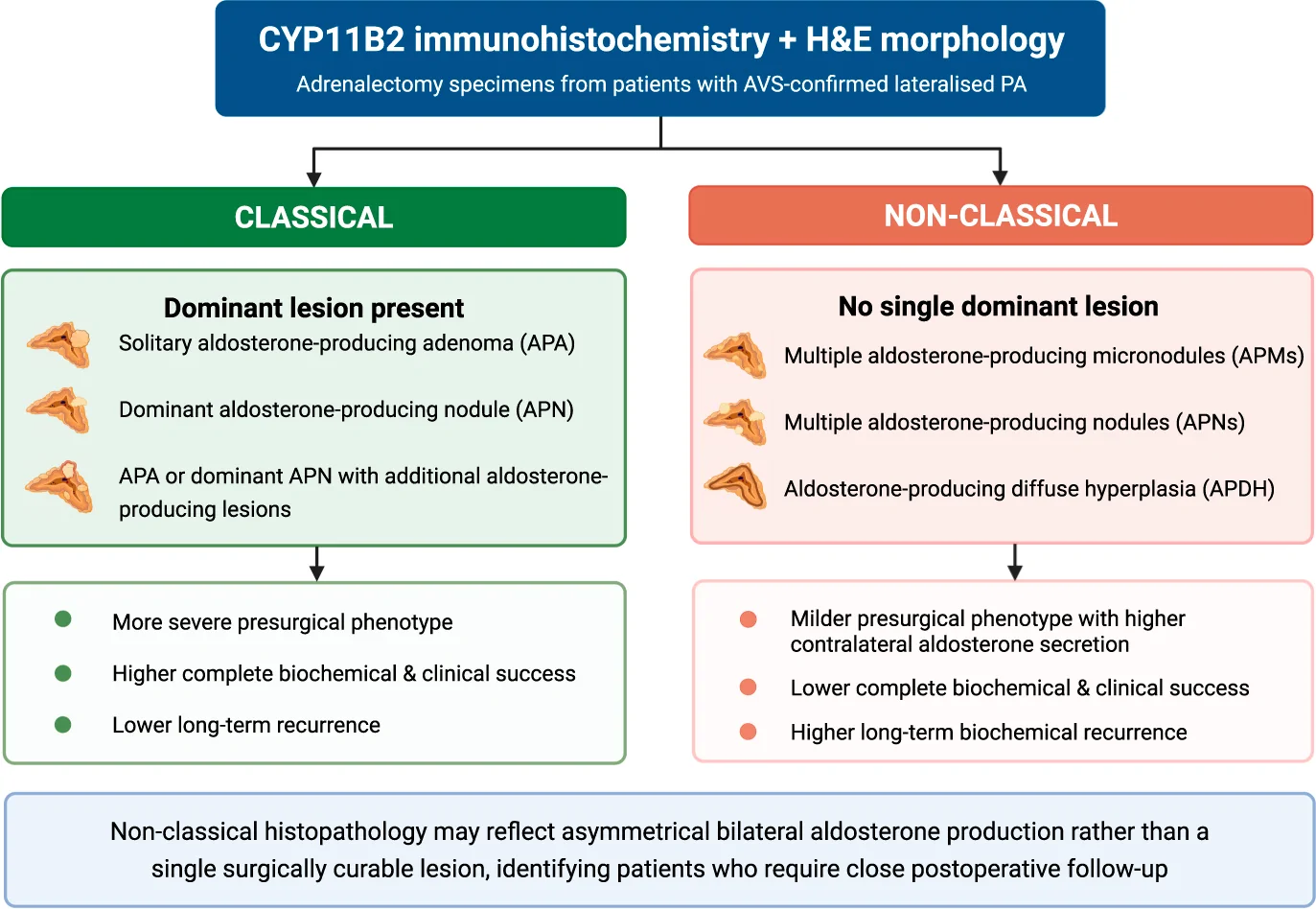
HISTALDO classification of lateralised primary aldosteronism. CYP11B2 immunohistochemistry combined with H&E morphology assigns resected adrenals to classical (a dominant lesion present: solitary APA, dominant APN, or APA/dominant APN with additional aldosterone-producing lesions) or non-classical (no single dominant lesion: APMs and/or APNs or aldosterone-producing diffuse hyperplasia) categories. Compared with non-classical histology, classical lesions are associated with a more severe presurgical phenotype, higher complete biochemical and clinical success, and lower long-term recurrence; non-classical histology is associated with a milder presurgical phenotype, higher contralateral aldosterone secretion, lower complete biochemical and clinical success and higher long-term biochemical recurrence. Non-classical histopathology may reflect asymmetrical bilateral aldosterone production rather than a single surgically curable lesion, identifying patients who require close postoperative follow-up. Created in BioRender https://BioRender.com/urexk8g.

The histopathology of primary aldosteronism (HISTALDO) consensus was developed by an international group of adrenal pathologists, endocrine clinicians and researchers to standardise the nomenclature and classification of aldosterone-producing lesions and related adrenal histopathological features in resected glands from patients with lateralised PA (19). This framework was subsequently endorsed by the 2022 WHO classification of adrenal cortical tumours (20). It uses CYP11B2 immunohistochemistry, read alongside routine haematoxylin and eosin (H&E) morphology, for the classification of aldosterone-producing regions into four lesion types: an APA, an aldosterone-producing nodule or micronodule (APN or APM), or aldosterone-producing diffuse hyperplasia (APDH). A practical application lies in the stratification of these lesions into classical histopathology of lateralised PA, defined by an APA or a dominant APN, with or without additional minor aldosterone-producing lesions; and non-classical lateralised PA, which encompasses multiple APMs, multiple APNs or APDH and the absence of a dominant lesion (19) (Fig. 2).

The relationship between this histological dichotomy and surgical outcome is clarified by AVS findings. Patients who do not achieve biochemical remission show a higher prevalence of multiple aldosterone-producing lesions (non-classical histology of lateralised PA) and a lower prevalence of a solitary APA, together with lower lateralisation indices (29, 40) and higher contralateral ratios at AVS (13, 40). In a multicentre series of patients operated on the strength of strong AVS lateralisation, roughly one in six were not biochemically cured, and within that subgroup multiple CYP11B2-positive nodules predominated (28). Histology therefore highlights that strong AVS lateralisation may reflect relative ipsilateral dominance rather than the absence of contralateral disease.

The clinical correlates of the classical *versus* non-classical categorisation are reproducible across cohorts and are summarised in Table 1. Counterintuitively, the more severe presurgical biochemical phenotype of classical histology is associated with better outcomes – a higher prevalence of complete biochemical (13, 14, 40) and clinical success (14) and lower long-term recurrence (41) – whereas non-classical histology, despite a milder presurgical phenotype, shows greater contralateral aldosterone secretion (13, 40), inferior surgical success (13, 14, 40) and a markedly higher prevalence of biochemical recurrence on extended follow-up (41). The mixed phenotype, in which a dominant lesion coexists with additional aldosterone-producing lesions, behaves as an intermediate (40). Thus, non-classical histology often reflects bilateral or multifocal aldosterone-producing disease, explaining why these patients may warrant closer biochemical surveillance after surgery even after initial biochemical remission.

**Table 1:** Histopathological category as a determinant of postoperative outcome in lateralised primary aldosteronism.

Outcome measure	Classical	Non-classical	Note	References
Complete biochemical success	97–98%	67–69%	Higher with classical histology	13, 14
Complete clinical success	34%	11%	Higher with classical histology	14
Postoperative antihypertensive medication burden (DDD)	0.00 (0.00–1.00)	1.00 (0.00–2.06)	Classical or mixed phenotypes associated with fewer medications	40, 14
Long-term biochemical recurrence after initial remission	14%	60%	Recurrence among patients with initial complete biochemical success over extended follow-up	
Contralateral adrenal-to-peripheral aldosterone ratio	2.0 (1.1–3.1)	3.8 (1.7–6.5)	Higher in non-classical histology, consistent with greater contralateral aldosterone production	13
Key interpretation	More consistent with dominant unilateral aldosterone production	More consistent with micronodular aldosterone production and asymmetrical bilateral PA	Classical histology predicts greater likelihood of remission	13, 14

Values are from studies applying CYP11B2-guided histopathological classification according to HISTALDO (19) and postoperative outcome assessment according to PASO (26). Complete biochemical and clinical success refer to short-term postoperative assessment unless otherwise stated. Long-term biochemical recurrence refers to recurrence among patients with initial complete biochemical success at 6–12 months. Contralateral adrenal-to-peripheral aldosterone ratio is the ratio of absolute aldosterone concentration in the contralateral adrenal vein to that in a peripheral vein; values are reported as median (IQR). Antihypertensive medication burden is expressed as median defined daily dose (DDD) (IQR), where DDD is the assumed average maintenance dose per day for a drug used for its main indication in adults (https://www.who.int/tools/atc-ddd-toolkit/about-ddd).

### From binary disease to biological continuum

The clinical dichotomy between lateralised and bilateral PA is useful for directing treatment but obscures the continuity of the underlying disorder. A series of physiological studies has reframed PA from a threshold-defined diagnosis towards a continuum of renin-independent aldosterone production. In normotensive individuals with suppressed renin, urinary aldosterone excretion during oral sodium loading varied continuously with urinary potassium excretion, angiotensin II-stimulated aldosterone and plasma renin activity; 14% reached confirmatory biochemical criteria for PA despite normal blood pressure (42). In a larger cross-sectional study spanning normotension to resistant hypertension, non-suppressible, renin-independent aldosterone production was present across every blood pressure category, increased with blood pressure severity and kaliuresis and was accompanied by lower serum potassium; conventional screening with the aldosterone-to-renin ratio was relatively insensitive for detecting biochemically overt PA (43). Taken together, these data support the view that clinically recognised PA lies at the severe end of a broader low-renin mineralocorticoid phenotype, rather than being entirely separable from milder renin-independent aldosterone production.

Subsequent studies indicate that this continuum is neither reducible to the choice of diagnostic threshold nor captured by renin independence alone. In an international low-renin cohort spanning normal blood pressure to severe or resistant hypertension, non-suppressible aldosterone after saline suppression varied continuously with blood pressure and correlated with adrenocorticotropic hormone (ACTH)-stimulated aldosterone production (44). The same pre-categorical signal is detectable in prospectively recruited normotensive participants, in whom the magnitude of renin-independent aldosterone production formed a graded spectrum associated with higher daytime ambulatory systolic blood pressure, even within the normotensive range, and with greater urinary potassium excretion (45). The continuum is therefore layered, with ACTH-mediated aldosterone responsiveness and lateralised disease detectable below conventional diagnostic thresholds and before overt hypertension is established.

**Figure 3:**
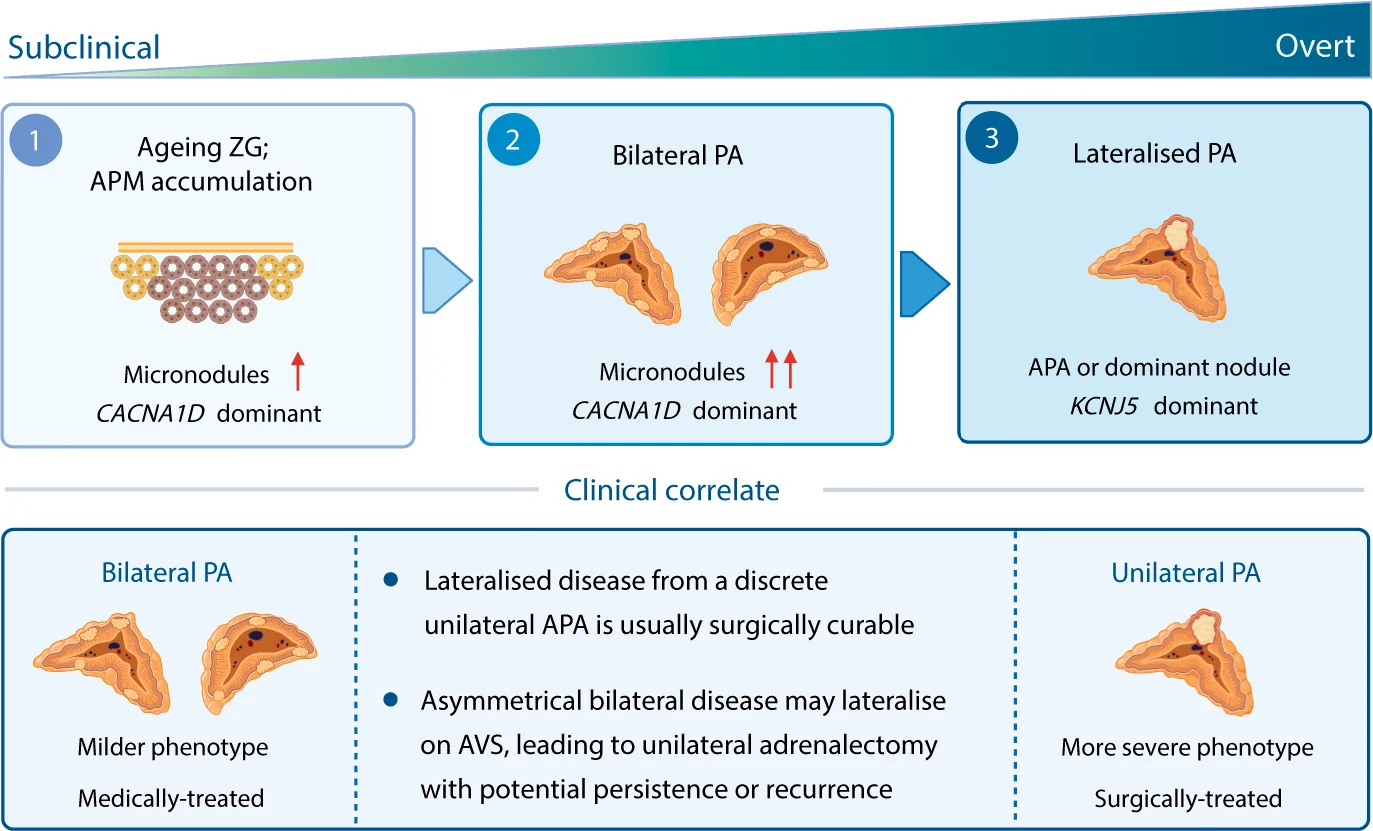
Primary aldosteronism as an evolutionary spectrum. Autonomous aldosterone production ranges from subclinical, renin-independent aldosteronism in the ageing zona glomerulosa, through accumulation of *CACNA1D*-dominant aldosterone-producing micronodules and *CACNA1D*-dominant bilateral disease, to overt lateralised PA, typically associated with a *KCNJ5*-dominant APA or dominant nodule. Clinically, bilateral PA presents with a milder phenotype and is medically treated, whereas unilateral PA presents with a more severe phenotype and is surgically treated; lateralised disease arising from a discrete unilateral APA is usually surgically curable, whereas asymmetrical bilateral disease may lateralise on AVS and lead to unilateral adrenalectomy with potential persistence or recurrence. APA, aldosterone-producing adenoma; APM, aldosterone-producing micronodule; AVS, adrenal vein sampling; *CACNA1D*, gene encoding the Ca_V_1.3 L-type voltage-gated calcium channel; *KCNJ5*, gene encoding the potassium inwardly rectifying channel subfamily J member 5; PA, primary aldosteronism. Created in BioRender https://BioRender.com/bxh0kq3.

This continuum has a correlate in adrenal tissue. In normal adrenal glands, ageing is accompanied by a decline in normal zona glomerulosa CYP11B2 expression and an increasing burden of APMs, paralleled clinically by progressively lower renin activity and an increasing aldosterone-to-renin ratio during high-sodium balance (11). A substantial proportion of these micronodules, roughly one-third in normal adrenals, carry somatic aldosterone-driver mutations, most often in *CACNA1D* and rarely in *KCNJ5*, a mutation profile overlapping but distinct from that of APAs (46, 47, 48). In a small series of patients with bilateral PA, aldosterone-producing diffuse hyperplasia was present in only a minority of glands. Instead, all examined adrenals harboured APMs, which were more numerous and larger than those in normal adrenals; somatic *CACNA1D* mutations were identified in 58% of sequenced micronodules (12). Physiological zonation therefore gives way to discontinuous, micronodular CYP11B2 expression enriched for aldosterone-driver mutations, creating a tissue context in which discrete, radiologically evident APAs may emerge in some glands (Fig. 3).

The spectrum model also explains why aldosterone excess can recur after an apparently curative adrenalectomy. Even a solitary APA is removed against the background of a bilateral adrenal tendency to aldosterone dysregulation. Excising it eliminates the principal source of aldosterone but not the potential of the contralateral gland to generate renin-independent aldosterone production over time, whether through expansion of lesions already present subclinically or the emergence of new ones. This propensity may sometimes be reflected before surgery by incomplete contralateral suppression on AVS. Consistent with this interpretation, contralateral suppression has been associated with better biochemical and clinical outcomes after adrenalectomy in several retrospective cohorts, although its definition varies and its predictive accuracy has been modest in some studies (49, 50, 51). Nevertheless, AVS captures adrenal function at a single time point and cannot exclude the subsequent emergence or expansion of renin-independent aldosterone production in the remaining gland.

### The genetics of aldosterone-producing adenomas

The somatic genetics of PA has been comprehensively reviewed elsewhere (15, 52); the features relevant here are those that connect genotype to the tissue spectrum described above and to surgical outcome. Somatic driver mutations are detectable in the large majority of APAs, and most act by sustaining intracellular calcium signalling, the central signal driving constitutive *CYP11B2* transcription. Most occur in ion channels or pumps that perturb calcium handling through membrane depolarisation, enhanced calcium entry or impaired calcium export. *KCNJ5* mutations, the most common driver (53), characterise larger, clear-cell, zona fasciculata-like adenomas, whereas *CACNA1D* and *ATP1A1* mutations tend to occur in smaller, eosinophilic, zona glomerulosa-like adenomas (54, 55); *ATP2B3*-mutated tumours are usually small but share some clear-cell features with *KCNJ5*-mutated APAs (56). *KCNJ5*-mutated APAs are also associated with higher circulating concentrations of the hybrid steroids 18-hydroxycortisol and 18-oxocortisol, reflecting the coexistence of zona fasciculata-like steroidogenic features with CYP11B2 expression. This distinctive steroid profile correlates with the clear-cell histopathological phenotype and may provide a circulating biomarker of *KCNJ5*-mutated APA, although its clinical utility for subtype classification requires further validation (57, 58, 59)

These genotype–phenotype associations extend to surgical prognosis and map onto the tissue spectrum set out above. *KCNJ5* mutations are associated with a higher incidence of postsurgical complete clinical (60, 61, 62) and biochemical (59) success, whereas *CACNA1D* mutations are associated with lower complete clinical success (63). This difference is consistent with the anatomical distribution of each driver. *CACNA1D* mutations predominate in APMs and in more micronodular patterns of aldosterone-producing tissue; consequently, a *CACNA1D*-mutated adenoma may be more likely to arise in a glandular context where additional aldosterone-producing lesions, including contralateral micronodular disease, contribute to residual aldosterone production after unilateral surgery. By contrast, *KCNJ5* mutations tend to define discrete adenomas whose resection is more often curative. This offers a genetic correlate of the postoperative persistence and recurrence described above, although the relationship between adenoma genotype and contralateral disease has not been systematically examined.

### Tissue omics and the developmental continuum of PA

Histology and somatic genotyping have defined the architecture and molecular drivers of aldosterone-producing lesions, establishing APAs and APMs as biologically active components of PA. What remains less clear is how aldosterone-producing cells are organised within the adrenal, how they relate to surrounding cortex and how microscopic lesions persist, diversify or progress. Single-cell, single-nucleus and spatial approaches add this complementary layer by mapping aldosterone-producing cell states onto tissue architecture and by placing APMs, APAs and adjacent cortical programmes within a broader developmental and spatial continuum (22). Tissue omics studies of PA adrenals are summarised in Table 2.

**Table 2:** Single-cell and spatial omics studies of aldosterone-producing cells in human adrenal tissue.

Method	Adrenal source	Lesions profiled	Driver gene in APAs (*n*)	Comparator	Key finding	Reference
Spatial metabolomics	PA adrenals	4 APMs, 4 APAs	*KCNJ5* (3), *CACNA1D* (1)	Paired cortex	APMs may evolve into APAs via a possible APM-to-APA transitional lesion	69
Spatial metabolomics	PA adrenals	136 APAs (132 genotyped)	*KCNJ5* (49), *ATP1A1* (9), *ATP2B3* (6), *CACNA1D* (11), *CTNNB1* (2), *PRKACA* (2), NMD (53)	Paired cortex	APAs exhibit genotype-specific metabolomic profiles	70
Spatial metabolomics	PA adrenals	27 APMs, 6 APAs	*KCNJ5* (3), *ATP1A1* (1), NMD (2)	Paired cortex	APM subgroups identified, one converging metabolically on APAs	21
scRNA-seq	Normal adrenals	2 APMs	Not reported	Adjacent ZG cells	APMs share transcriptional states with ZG cells, supporting a ZG-derived origin	71
scRNA-seq	PA adrenals	3 APAs	Not reported	Paired ZG cells	Resolved APA cellular heterogeneity relative to adjacent normal cortex	72
Spatial transcriptomics	PA adrenals	7 APAs	*KCNJ5* (4), *CACNA1D* (1), NMD (2)	Paired cortex	Genotype-dependent spatial heterogeneity and metabolic profiles linked to tumour expansion	22
scRNA-seq	PA adrenals	3 APAs	*KCNJ5* (3)	2 NFAs	Defined APA cell states and a fasciculata-like signature in *KCNJ5*-mutated tumours	73
scRNA-seq + spatial transcriptomics	PA adrenals	scRNA-seq: 8 APAs; spatial transcriptomics: 4 APAs	scRNA-seq: *KCNJ5* (8); spatial transcriptomics: *KCNJ5* (4)	Paired ZG cells	Single-cell atlas of *KCNJ5*-mutated APAs resolving cell-state composition and microenvironment	64
scRNA-seq	PA adrenals	3 APAs	Not reported	3 normal adrenals	Single-cell atlas of APAs revealing metabolic reprogramming and tumour microenvironment remodelling	74
scRNA-seq + spatial transcriptomics	PA adrenals	3 APAs	*KCNJ5* (3)	Paired cortex	Functional heterogeneity of CYP11B2^+^ cells and macrophage-mediated tumour crosstalk in *KCNJ5*-mutated APAs	75
sc/snRNA-seq + spatial transcriptomics	PA adrenals	sc/snRNA-seq: 13 APAs, 2 APMs; spatial transcriptomics: 3 APAs, 17 APMs	sc/snRNA-seq: *KCNJ5* (12), *ATP1A1* (1); spatial transcriptomics: *KCNJ5* (1), *ATP1A1* (1), *CACNA1D* (1)	Paired ZG cells / paired cortex	Evolutionary trajectories and cell density-dependent ferroptosis mechanisms in APAs	65

Studies are listed chronologically and restricted to those profiling APMs or APAs in human adrenal tissue by single-cell, single-nucleus or spatially resolved transcriptomics or metabolomics. Lesions profiled, number and type of aldosterone-producing lesions analysed; driver gene, gene carrying the aldosterone driver mutation in APA (*n*, number of APA with indicated driver mutation); comparator, reference tissue or cell population; peritumoural adrenal cortical tissue from the same adrenalectomy specimen as the APA; adjacent ZG cells, CYP11B2-negative ZG cells adjacent to the APM; paired ZG cells, ZG cells from the same adrenalectomy specimen as the APA; normal adrenals, histologically normal adrenal tissue obtained during incidental adrenalectomy at radical nephrectomy. APA, aldosterone-producing adenoma; APM, aldosterone-producing micronodule; NFA, non-functioning adenoma; NMD, no mutation detected; PA, primary aldosteronism; scRNA-seq, single-cell RNA sequencing; sc/snRNA-seq, single-cell/single-nucleus RNA sequencing; ZG, zona glomerulosa.

Single-cell and spatial transcriptomic studies support an inferred developmental path from zona glomerulosa cells through APMs to APAs. These aldosterone-producing cell states overlap transcriptionally but are not identical. Reprogramming appears to begin in a stress-responsive, progenitor-like state and then proceeds towards steroidogenic fates, with some tumours additionally acquiring cortisol-producing and stromal-like programmes (64, 65). This does not imply that every lesion follows a single linear route, but it does suggest that many aldosterone-producing lesions are not separate entities. Rather, they represent different stages or outcomes of a shared adrenal programme.

**Figure 4:**
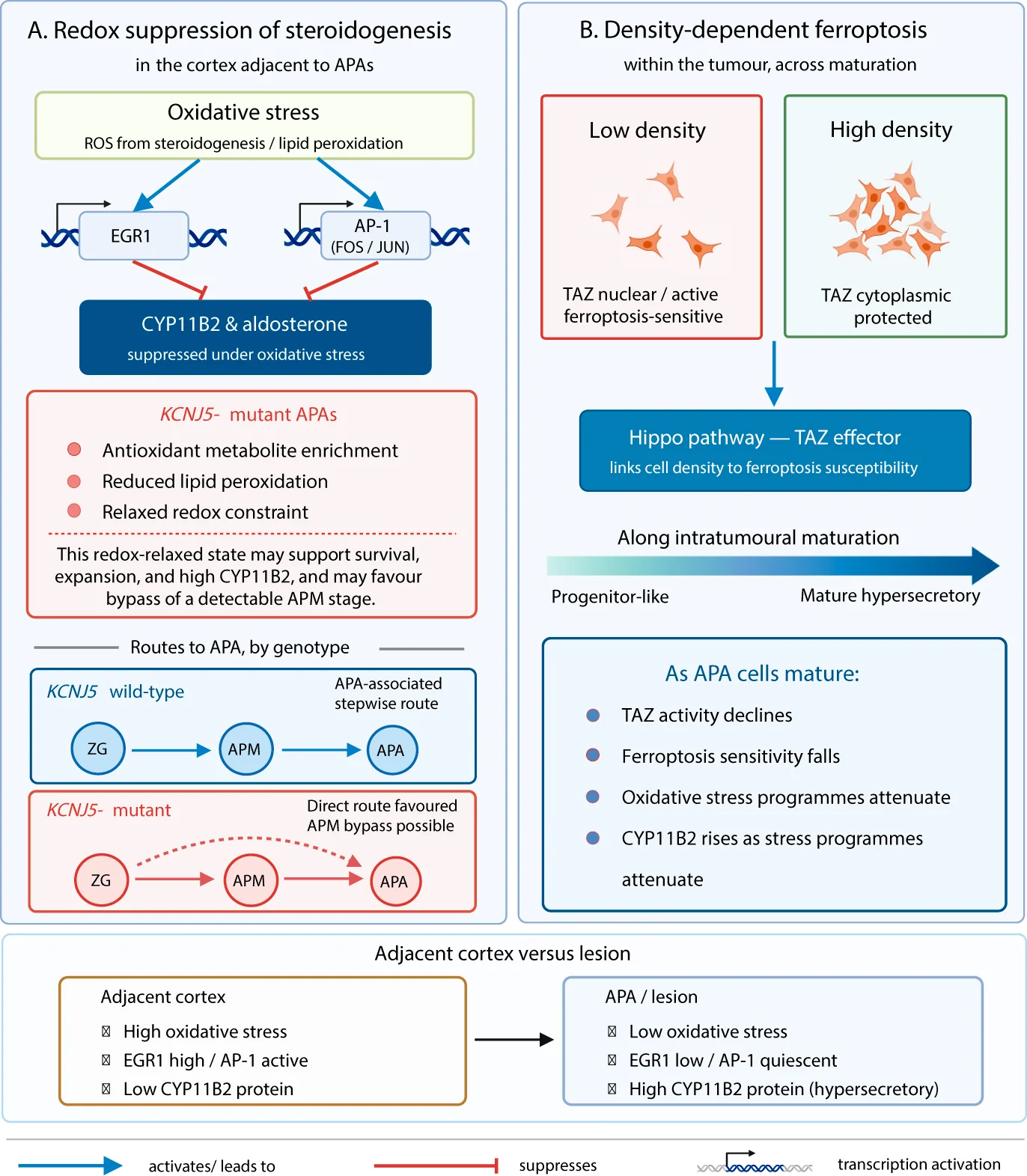
Redox and ferroptosis control of steroidogenesis and lesion expansion. (A) Oxidative stress arising from steroidogenesis and lipid peroxidation activates stress-responsive transcriptional programmes, including EGR1 and AP-1 (FOS/JUN), which suppress *CYP11B2* expression and aldosterone production. This programme is prominent in cortex adjacent to aldosterone-producing adenomas – where oxidative stress is high, EGR1 is high, AP-1 is active and CYP11B2 protein is low – but is attenuated within the lesion, where oxidative stress is low, EGR1 is low, AP-1 is quiescent and CYP11B2 protein is high. In *KCNJ5*-mutant APAs, antioxidant metabolite enrichment and reduced lipid peroxidation are consistent with a relaxed redox constraint. This redox-relaxed state may support cell survival, expansion and high *CYP11B2* expression and may favour a route to APA formation that bypasses a detectable APM stage. Accordingly, whereas the *KCNJ5*-wild-type route to APA is stepwise (ZG→APM→APA), the *KCNJ5*-mutant route may proceed directly, with possible APM bypass. (B) Cell density provides a second regulatory axis during intratumoural maturation, linked through the Hippo pathway effector TAZ. At low density, TAZ is nuclear and active, and cells remain ferroptosis-sensitive; at high density, TAZ is cytoplasmic and cells are protected. As APA cells mature from a progenitor-like to a mature hypersecretory state, TAZ activity declines, ferroptosis sensitivity falls, oxidative-stress programmes attenuate and *CYP11B2* expression rises. Together, these processes suggest a model in which APA progression is supported by escape from oxidative-stress-mediated suppression of steroidogenesis and acquisition of density-dependent resistance to ferroptosis. Blue arrows denote activation, and red bars denote suppression. APA, aldosterone-producing adenoma; APM, aldosterone-producing micronodule; AP-1, activator protein 1 (a dimeric transcription factor complex); CYP11B2, cytochrome P450 family 11 subfamily B member 2 (aldosterone synthase); EGR1, early growth response 1 (a stress-induced transcription factor regulating cell survival and tissue remodelling); FOS/JUN, basic leucine zipper proteins that form the functional AP-1 complex; Hippo, upstream tumour-suppressive kinase cascade that inhibits YAP/TAZ; *KCNJ5*, gene encoding the potassium inwardly rectifying channel subfamily J member 5; TAZ, transcriptional co-activator with PDZ-binding motif; ZG, zona glomerulosa. Created in BioRender https://BioRender.com/gkbgboj.

This programme is strongly shaped by genotype. *KCNJ5* mutations are frequent in overt APAs but rare in APMs, where *CACNA1D* mutations are more common (12, 65). Integrated single-cell and spatial analyses offer a way to reconcile this apparent paradox by supporting biologically plausible routes to adenoma formation, including a stepwise route through an APM-like intermediate, seen in both *KCNJ5*-mutated and *KCNJ5*-wild-type tumours, and a more direct zona glomerulosa-to-APA route seen predominantly in *KCNJ5*-mutated tumours (65) (Fig. 4). *KCNJ5*-wild-type lesions therefore appear more dependent on an APM-like intermediate state, whereas *KCNJ5*-mutated lesions may bypass a detectable micronodule stage. This provides a plausible biological explanation for why the most common driver of large APAs is under-represented in APMs.

One proposed mechanism for this divergence is a genotype-dependent redox constraint on lesion growth. Steroidogenic activation imposes oxidative stress, and *KCNJ5*-wild-type tumours show accumulation of prooxidant and proferroptotic lipid species, consistent with a growth-limiting stress response that restrains expansion. By contrast, *KCNJ5*-mutated tumours reprogramme towards antioxidant defence and show lower oxidative lipid damage (22, 65, 66). In this model, sparse nascent clusters would be most vulnerable to ferroptotic stress, whereas increasing cell density and tumour maturation are associated with Hippo pathway-related changes in TAZ (transcriptional coactivator with PDZ-binding motif) localisation and activity, reduced ferroptosis susceptibility and attenuation of oxidative-stress-related programmes (65). *KCNJ5*-mutated cells, being relatively redox-buffered, may therefore survive and expand more freely, offering a coherent explanation for their tendency to form larger, more florid adenomas (53).

This redox model also connects lesion growth with aldosterone output. Oxidative-stress markers are lower in APAs than in adjacent cortex, most markedly in *KCNJ5*-mutated lesions, while lipid-peroxidation stress represses CYP11B2 and aldosterone production through the redox-responsive factors EGR1 and AP-1 (FOS/JUN) (67, 68). The EGR1/AP-1 stress-response programme is downregulated or functionally restrained in the tumour but retained in the surrounding cortex. The inverse relationship between oxidative stress and steroidogenesis may therefore help explain why some adjacent cortical regions retain stress-responsive EGR1/AP-1 activity and remain CYP11B2-negative, whereas established tumour regions suppress this programme and sustain aldosterone production (Fig. 4).

This genotype-dependent divergence is also evident at the transcriptional level. Two recurring CYP11B2-positive programmes have been described, one zona glomerulosa-like programme present across genotypes and a second zona fasciculata/reticularis-like programme that predominates in *KCNJ5*-mutated tumours (22). Established APAs are also spatially organised lesions, with aldosterone-producing cells embedded in distinct immune-stromal microenvironments. Across transcriptomic and spatial studies, they show altered immune composition, including immunosuppressive myeloid populations whose abundance and signalling differ between micronodules and adenomas (65). In *KCNJ5*-mutated APAs specifically, lipid-associated macrophages are enriched and correlate with intratumoural cortisol synthesis and tumour size, linking the myeloid niche to the cortisol co-secretion seen in a subset of these tumours (64). Whether this niche actively supports hormone production and tumour growth or instead reflects tumour maturation, remains unresolved.

Taken together, these studies suggest that genotype, redox state and tissue context shape whether aldosterone-producing lesions remain microscopic or expand into dominant APAs. *KCNJ5*-mutated tumours may therefore represent a redox-adapted route to discrete, surgically remediable adenomas, whereas micronodular or bilateral disease reflects a more diffuse aldosterone-producing process with greater risk of persistent or recurrent aldosteronism after adrenalectomy.

### Clinical implications and future directions

Assessment of treatment response in PA should distinguish control of aldosterone excess from reversal of established hypertension. PASO and PAMO formalise this distinction in complementary clinical settings (26, 37). After adrenalectomy, removal of the dominant aldosterone source usually corrects the biochemical phenotype of AVS-confirmed lateralised PA, whereas blood pressure normalisation is less frequent and is influenced by age, sex, preoperative antihypertensive burden, coexisting primary hypertension and the reversibility of aldosterone-mediated vascular or renal injury. PAMO extends the same principle to medically treated PA, where renin recovery provides a measurable target of adequate MR blockade, while complete clinical response remains uncommon under current practice. The clinical aim is therefore not only to reduce blood pressure but to determine whether aldosterone excess has been removed, adequately blocked or remains biologically active.

This distinction is important for subtype interpretation. Strong AVS lateralisation should be read as dominance rather than proof of true unilateral disease, because asymmetrical bilateral PA can persist despite robust lateralisation and may require long-term biochemical surveillance (28). In this context, CYP11B2-guided histopathology extends beyond morphological classification by identifying patients at greater risk of persistent or recurrent aldosteronism who may benefit from closer postoperative biochemical surveillance (13, 41). Genotype may add a further layer of interpretation by linking clinical phenotype to lesion biology. *KCNJ5* mutations are typically associated with dominant APAs and a surgically remediable phenotype (60, 61), whereas *CACNA1D*-mutated APMs or APM-rich histology point to a micronodular pattern of disease that may be bilateral or leave residual aldosterone production after removal of the dominant gland (12).

Tissue omics adds a further translational layer, although the adrenal samples analysed in these studies are necessarily obtained at surgery and are not yet used prospectively to guide clinical management. Its value is to define the cellular states, spatial relationships and immune-stromal programmes that underlie the phenotypes already recognised by AVS, CYP11B2-guided histopathology and genotype. By mapping the cellular continuum that underlies PA, these studies provide a biological rationale for why PA behaves as a spectrum rather than a unilateral-bilateral binary. This may ultimately inform preoperative decision-making as biomarkers emerge that identify discrete surgically remediable disease, multifocal aldosterone-producing tissue or risk of persistent or recurrent aldosteronism before treatment decisions are made.

These translational implications also define the next questions. The upstream signals that establish or maintain the low-oxidative-stress state of *KCNJ5*-mutated APAs are still unclear, and whether the macrophage niche actively supports aldosterone production, cortisol co-secretion or tumour survival has not been directly tested. Biomarkers that predict non-classical histopathology, bilateral aldosterone-producing tissue or future persistent or recurrent aldosteronism before surgery would be of clinical value. Finally, ferroptosis, redox adaptation and microenvironmental signalling are not therapeutic targets in PA. At present, they provide a conceptual entry point for intervention rather than a treatment strategy, but they indicate the type of biology that may eventually distinguish patients most likely to benefit from surgery, intensified medical therapy or closer surveillance.

## Conclusion

PA is best understood as a dynamic spectrum of autonomous aldosterone production, encompassing subclinical renin-independent aldosteronism, age-related APM accumulation, asymmetrical bilateral disease and clinically overt dominant APAs. PASO, PAMO and HISTALDO have refined how treatment response and tissue phenotype are interpreted, while single-cell biology, spatial transcriptomics and spatial metabolomics have begun to connect these clinical categories to the cellular states, tissue microenvironments and metabolic constraints within which aldosterone-producing lesions arise, persist or expand. Together, these advances shift PA from a binary model of unilateral *versus* bilateral disease towards a continuum in which dominance, multifocality and recurrence reflect different expressions of shared adrenal biology. From bench to bedside, the central message is that PA is common, biologically complex and clinically modifiable. The present challenge is to diagnose it earlier, treat aldosterone excess more completely and, in surgically treated patients, use CYP11B2-guided histopathology and molecular context to identify those at greatest risk of persistent or recurrent aldosteronism and residual cardiovascular risk.

## Declaration of interest

The authors declare that there is no conflict of interest that could be perceived as prejudicing the impartiality of this review.

## Funding

The work of TAW was supported by the Else Kröner-Fresenius-Stiftung (2024-EKSE.191) and by the Deutsche Forschungsgemeinschaft (DFG), project numbers 570979695 (WI 5359/4-1) and 314061271-TRR 205/B15 and B19 within ‘The Adrenal: Central Relay in Health and Disease’. The work of JW was supported by the China Scholarship Council.

## Author contribution statement

JW and TAW wrote the manuscript and prepared the figures and tables.

## Ethics statement

This is a review article and did not involve new studies with human participants, human tissue, animals or patient-identifiable data.
